# *Lactobacillus plantarum* L15 Alleviates Colitis by Inhibiting LPS-Mediated NF-κB Activation and Ameliorates DSS-Induced Gut Microbiota Dysbiosis

**DOI:** 10.3389/fimmu.2020.575173

**Published:** 2020-10-02

**Authors:** Peng Yu, Chuxin Ke, Jiaxin Guo, Xiuling Zhang, Bailiang Li

**Affiliations:** ^1^College of Food Science, Northeast Agricultural University, Harbin, China; ^2^Key Laboratory of Dairy Science, Ministry of Education, Northeast Agricultural University, Harbin, China

**Keywords:** *Lactobacillus plantarum*, DSS-colitis, gut microbiota, lipopolysaccharide (LPS), NF-κB signaling

## Abstract

Previous studies have suggested that the *Lactobacillus plantarum* bacteria strain could be effective in ulcerative colitis (UC) management. However, its effects are strain-specific and the related mechanisms for its attenuating effects on UC remain unclear. This study aimed to elucidate the underlying mechanisms for the protective effect of *L. plantarum* on UC. Firstly, 15 *L. plantarum* strains were screened for potential probiotic characteristics with good tolerance to simulated human gastrointestinal transit and adhesion. Secondly, the inflammatory response of selected strains to the Caco-2 cells induced by lipopolysaccharide (LPS) was measured. Finally, an *in vivo* mouse model induced by dextran sulfate sodium (DSS) was used to assess the beneficial effects and likely action mechanisms the successfully screened *in vitro* strain, *L. plantarum* L15. *In vitro* results showed that *L. plantarum* L15 possessed the highest gastrointestinal transit tolerance, adhesion and reduction of pro-inflammatory abilities compared to the other screened strains. *In vivo*, high dose of *L. plantarum* L15 supplementation increased the body weight, colon length and anti-inflammatory cytokine production. Pro-inflammatory cytokine production, disease activity index (DAI) levels and myeloperoxidase (MPO) parameters decreased using this strain. In addition, *L. plantarum* L15 alleviated the histopathological changes in colon, modulated the gut microbiota, and decreased LPS secretion. The activities of this strain down-regulated the expression of TLR4 and MyD88 genes as well as genes associated with NF-κB signaling pathway. Our findings present *L. plantarum* L15 as a new probiotic, with promising application for UC management.

## Introduction

Ulcerative colitis (UC) is of increasing concern worldwide, with inflammatory characteristics between the rectum and colon sections of sufferers (1). Although the exact causative factors are still unclear at present, several hypotheses have linked UC to genetic variations, environmental changes, and immunomodulatory factors (2). Conventionally, UC can be treated with a number of medications, including 5-aminosalicylic acid drugs, steroids, and immunosuppressants. These therapies have a number of drawbacks, ranging from unpleasant reactions to high cost especially for patients who may need them for a long time frame (3). Therefore, it becomes important to find a healthy and effective way to manage UC.

Microbes in the human intestinal system give tremendous metabolic and health-related support to the body, thus leading to the postulation that the gut microbiota is another body “organ” (4). Cumulative studies on the gut microbiota have informed a research position that this “organ” could be a target for therapeutic interventions as it has been linked with health and well-being (5, 6). In addition, next-generation sequence (NGS) techniques and *in vivo* reports have recently demonstrated that imbalances in microbiota profiles have been found in highly prevalent diseases, such as inflammatory bowel diseases (IBD), irritable bowel syndrome (IBS) or colorectal cancer, among others (6–9). Furthermore, the decrease in the relative abundance of healthy gut microbiome phyla like Firmicutes and a corresponding increases in Proteobacteria has been observed in both Crohn's Disease (CD) and UC gut microbiomes (10–12). Probiotic bacterial strains, mainly of the *Lactobacillus* genus, have been used to treat gut dysbiosis incidences and as such, can be a possible alternative in UC treatment. Some species from the *Lactobacillus* genera can regulate cytokine activities and improve integrity of the gut barrier (13). Furthermore, some species have been reported to have UC-alleviating effects by reducing the production of pro-inflammatory factors, and restoring the balance of the gut microbiota (14–16).

A number of studies involving different *L. plantarum* strains have demonstrated *in vitro* and *in vivo* beneficial effects in UC therapy through normalizing disease activity index (DAI) score, significantly suppressing the expression of pro-inflammatory factors (TNF-α and IL-6), modulating gut microbiota, halting intestinal cell apoptosis as well as inhibiting activation of the NF-κB signaling pathway (17–20). Thus, *L. plantarum* supplementation could be a fascinating strategy for the patients suffering from UC. However, there is little information about the UC-alleviating mechanisms of *L. plantarum* strains. In the present study, we selected a potential probiotic *L. plantarum* L15 strain with good gastrointestinal transit tolerance, adhesion, and reduction of pro-inflammatory abilities and further investigated the effect and the mechanism of *L. plantarum* L15 supplementation in an *in vivo* dextran sulfate sodium-induced colitis model, which has been widely used as a representative UC model, because of its simplicity and many similarities with human UC condition (21). DSS is a highly water-soluble compound which is toxic for the colonic epithelial cells and causes defects in the epithelial barrier integrity, resulting in increased colonic epithelial permeability (22). The aim of this study was to determine whether *L. plantarum* L15 supplementation can ameliorate DSS-induced UC by inhibiting LPS-mediated NF-κB activation and regulate DSS-induced gut microbiota dysbiosis. It is anticipated that this study will enhance our understanding regarding the use of *L. plantarum* L15 as a potential probiotic against UC in functional foods and supplements formulation.

## Materials and Methods

### Bacterial Strain and Culture

A total of 15 *Lactobacillus plantarum* strains (L11, L12, L13, L14, L15, L16, L17, L18, L19, L110, L111, L112, L113, L114, and L115) were isolated from yak yogurt in Gansu Province, China and identified by 16S rDNA similarity analysis. All strains were deposited in the Key Laboratory of Dairy Science (KLDS) of the Northeast Agricultural University (NEAU), Ministry of Education, China. Strains were anaerobically incubated in deMan Rogosa and Sharpe (MRS) broth (Hopebio Company, China) at 37°C for 24 h and sub-cultured twice prior to the experiment.

### Tolerance to Simulated Human Gastrointestinal Transit (GIT)

The tolerance to simulated human GIT of the 15 *Lactobacillus* strains was evaluated according to the method described by Maragkoudakis et al. (23). Briefly, the cells were harvested by centrifugation at 10,000 × g for 10 min at 4°C after incubation at 37°C for 18 h. Then they were adjusted to 10^9^ CFU/mL and subjected in simulated gastric juice for 3 h and small intestinal juice for 4 h. The microorganisms were harvested by centrifugation at 10,000 × g for 10 min at 4°C and transferred from simulated gastric juice to simulated small intestinal juice. The simulated gastric juice was prepared by supplementing pepsin (1:10,000, P8160, Solarbio Co., China) to normal saline (pH 3.0) at a concentration of 3 g/L. The simulated intestinal juice was prepared by adding 1 g/L trypsin (1:250, T8150, Solarbio Co., China) to normal saline (pH 8.0) to a concentration of 3 g/L bile salt (LP0055, Thermo Fisher Scientific Co., UK). All the prepared juices were filter-sterilized using 0.22 μm membrane filters. Viable cells were counted by plate colony counting method at 0 (N0) and 7 h (N1) to determine tolerance levels after incubation on MRS agar at 37°C for 48 h. The experiment was repeated in triplicate. The survival rate was calculated as follows:

$$\begin{array}{l} \text{Survival rate }\left(\text{\%}\right)=\frac{\text{lg CFU }{\text{N}}_{1}}{\text{ lg CFU }{\text{N}}_{0}}\times \;100\; \end{array}$$

### Adhesion to Caco-2 Cells

Caco-2 cells are purchased from the Cell Bank of the Chinese Academy of Sciences (Shanghai, China). They were then grown at 37°C in a humidified atmosphere of 95% air and 5% CO_2_. The cells were cultured in high-glucose Dulbecco's Modified Eagle's Medium (DMEM, Gibco, USA) supplemented with 10% fetal bovine serum (Gibco, USA) and 1% penicillin/streptomycin (Gibco, USA).

The adhesive ability of the strains using Caco-2 cells were determined as previously described with slight modifications (24). The Caco-2 cells were seeded at 10^5^ cells/well in a 12-well-plate and incubated at 37°C for 24 h. One day before the experiments, medium was replaced by DMEM (without penicillin/streptomycin). The lactobacilli cells were washed with phosphate buffer saline (PBS) thrice, and the cells were collected by centrifugation at 10,000 × g for 10 min followed by suspension with no glucose, no penicillin/streptomycin DMEM, adjusted to 10^8^ CFU (V0), and added in the wells. After being incubated for 2 h, the monolayer was washed three times with PBS to remove free and unattached bacterial cells. After that, 250 μL of 0.25% trypsin-EDTA (Gibco, USA) was added and incubated for 10 min, then the reaction was terminated using 250 μL of fetal bovine serum. Mixtures in each well was collected and used to evaluate the viable count of the strain (V1). The experiment was repeated in triplicate. Adhesion assays were performed in triplicate. The adhesive ability was expressed as:

$$\begin{array}{l} \text{Adhesion rate }\left(\text{\%}\right)=\frac{\text{V}1}{\text{V}0}\times 100\; \end{array}$$

### Induction of Inflammatory Response in Caco-2 Cells

The induction of inflammatory response in Caco-2 cells was performed following a previously described procedure (25). 250 ng/μl of lipopolysaccharide (LPS) solution (Sigma Chemicals, Bangalore) was used to initiate Caco-2 cells inflammatory response. Cells cultures with LPS were kept in a 5% CO_2_ incubator for 3 h. Cell cultures without LPS stimulation served as controls. After 4 h of treatment with 10^8^ CFU/mL cells (final concentration) of each *Lactobacillus* species. The treated Caco-2 cells along with strains were washed twice with PBS (pH 7.0) and the treated cells were detached from the tissue culture plates. Total RNA of Caco-2 cells was extracted using Trizol reagent (Life Technologies, USA). cDNA was obtained by reverse transcription with a PrimeScript RT reagent Kit with gDNA Eraser (Takara, Japan) according to the manufacturer's instructions. The mRNA expression of genes was performed by ABI 7500 fast real time PCR system (Applied Biosystems, USA) using SYBR Premix Ex Taq (Takara, Japan). The primers (Comate Bioscience Co., Ltd, China) used in this study are shown in Supplementary Table 1. The housekeeping gene (GAPDH) was used for normalization and data was analyzed by 2^−ΔΔCt^ method. The experiment was performed in six replications.

### Animals and Experimental Design

A total of 60 BALB/c mice (6 weeks old) were provided by the Vital River Laboratory Animal Technology Company (Beijing, China) and housed in a room under controlled environmental conditions at 22 ± 2°C, a relatively humidity of 40–60% and with a 12-h light/dark cycle. All conducted animal experiments in this study were approved by the Northeast Agricultural University Institutional Animal Care and Use Committee (Approval No: SRM-10).

The mice were placed in plastic cages for 1 week in order to acclimatize prior to the experiments and provided with normal chow diet and water *ad libitum*. They were randomly assigned to 5 groups (*n* = 12). Firstly, the control group was given distilled water, the DDS-induced colitis group (DSS) was received 3.5% (w/v) DSS (36–50 kDa, MP Biomedicals Ltd., Santa Ana, U.S.A.) in drinking water for 7 days. Then, the low-dose (LD) group and the high-dose (HD) group were given 1 × 10^9^ or 1 × 10^10^ CFU/mL *L. plantarum* L15 (1 mL/100 g body weight) (26, 27) by oral gavage once daily for a period of 28 days, respectively. Bacteria were harvested by centrifugation at 5,000 × g for 10 min. Cell pellets were then washed twice with sterile normal saline (9 g of Nacl per liter (0.9%) solution) and re-suspended at the concentration (1 × 10^9^ or 1 × 10^10^ CFU/mL) in sterile normal saline. The control and DSS groups were given equal volumes of sterile normal saline as no-treatment control and negative control, respectively. The positive control group (PC) was administered at a concentration of 30 mg/mL of salicylazosulfapyridine with the same volume of sterile normal saline (28).

### Growth Indices

During the experiment, the body weight, stool consistency, and blood in the stool of mice were recorded. Disease activity index (DAI) was scored based on an average score of body weight change, stool consistency, and hemoccult bleeding from a previous scoring system (Supplementary Table 2) (29, 30). The measurement was conducted in six replications.

### Histopathologic Analysis

Histopathologic analysis was performed as described in the previous research (19). Briefly, To observe colon histopathological changes after different treatments, colon samples were fixed in 10%-buffered formalin solution, dehydrated in ethanol, embedded in paraffin, cut into 5 μm sections. They were then stained using standard hematoxylin-eosin (HE) and then observed under a light microscope. Colon samples were observed at ×200 and ×400 magnification, and the histopathology changes were determined using a scoring system taking into account the severity of inflammation (from 0 to 3 as the maximum score), crypt damage (from 0 to 5 as the maximum score) and ulcerations (from 0 to 3 as the maximum score) as previously described (31). The experiment was performed in six replications.

### Biochemical Analyses

The colon samples were weighed and homogenized in nine volumes of ice-cooled PBS (pH 7.4), centrifuged at 5,000 × g for 10 min at 4°C to collect the supernatant, which was used for biochemical analyses. In this study, myeloperoxidase (MPO), superoxide dismutase (SOD), glutathione (GSH), and malondialdehyde (MDA) levels were determined using commercial detection kits (Nanjing Jiancheng Bioengineering Institute) following the manufacturer's protocol. Commercial enzyme-linked immunosorbent assay (ELISA) kits (Mei Lian Biotechnology Co., Ltd. Shanghai, China) were used to evaluate the concentrations of tumor necrosis factor alpha (TNF-α), interleukin-1β (IL-1β), IL-10, and IL-12. Finally, fecal and serum LPS contents were evaluated with the Limulus amebocyte lysate (LAL) assay kit (32). The measurement was conducted in six replications.

### Microbial Analysis of Cecal Contents

Bacterial DNA in the cecal contents of the control, DSS and HD groups were extracted using a QIAamp DNA stool mini kit (Qiagen, Dusseldorf, Germany) following the manufacturer's instructions. The V3-V4 region of the 16S rDNA were selected for generating amplicons by PCR technique. The primers were as follows: (forward primers: 5′-CCTACGGGNGGCWGCAG-3′, and reverse primers: 5′-GACTACHVGGGTATCTAATCC-3′). The PCR products were purified with QIAquick PCR Purification Kit (Qiagen, Dusseldorf, Germany), and quantified using a Qubit 2.0 Fluorometer (Thermo Fisher Scientific, Waltham, USA). Next-generation 16S rDNA sequencing was performed on an Illumina Miseq (Illumina, Santiago, USA). In all, 1,613,856 raw reads were obtained from cecal samples (Supplementary Table 3). The obtained data were merged using FLASH software V1.2.7 (33) and filtered with QIIME software V1.7.0 (34, 35). The chimera sequences were discarded by using the UCHIME algorithm to obtain the high-quality clean tags (36). The tags were clustered into distinct operational taxonomic units (OTUs) using Uparse software with a 97% sequence identity (37). They were annotated to taxonomic information by comparing to the GreenGene database using PyNAST software V1.2 (38). The phylogenetic tree was generated using MUSCLE software. OTUs abundance information was normalized using a standard of sequence number corresponding to the sample with the least sequences. QIIME software V1.7.0 were used to analyze α-diversity and β-diversity. The abundances of functional category of LPS biosynthesis in the KEGG pathway was predicted by Phylogenetic Investigation of Communities by Reconstruction of Unobserved States (PICRUSt) based on the 16S rRNA sequences (39). The measurement was conducted in five replications.

### Analyses of Gene Expression

Total RNA of colon tissue was extracted using Trizol reagent (Life Technologies, USA). The primers (Comate Bioscience Co., Ltd, China) used in this study are shown in Supplementary Table 4. Analyses using the qRT-PCR technique was performed as earlier described in this research, and the housekeeping gene (β-actin) was used for normalization. The western blotting technique was used to detect proteins involved in the colonic NF-κB signaling pathway as previously reported with slight modifications (20). Colon tissue samples were homogenized, centrifuged and the obtained supernatant analyzed by electrophoresis on 8–10% sodium dodecyl sulfate-polyacrylamide gel. Samples were then placed on a nitrocellulose membrane to measure the expressions of NF-κB p65, p-p65, IκB, p-IκB, and β-actin. The measurement was conducted in six replications.

### Statistical Analysis

All values are expressed as the mean ± standard deviation (SD). All experiments were performed at least three times using independent assays. The statistical significance of data comparisons was determined using one-way analysis of variance, followed by Duncan's multiple range test of the statistical software package Social Science version 22.0 (SPSS Inc., Chicago, IL, U.S.A.). Values of *P* < 0.05 indicated a significant difference.

## Results

### *In vitro* Probiotic Characteristics

**Figure 1:**
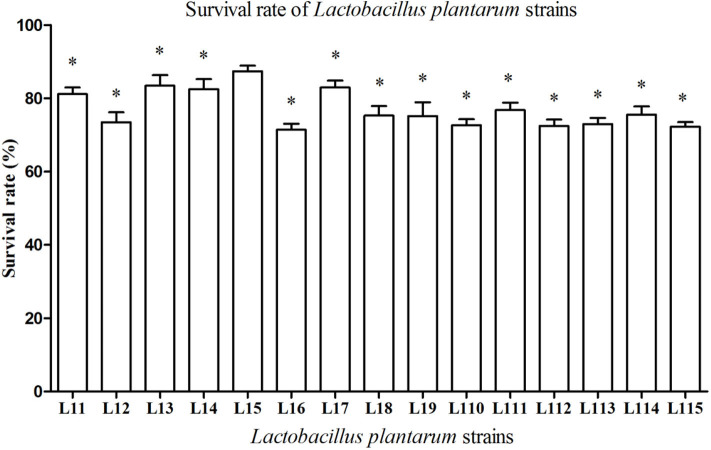
Tolerance of *Lactobacillus plantarum* strains to simulated gastrointestinal juices. Values are mean ± SD (*n* = 3 independent experiment). *P < 0.05: significantly different compared with *L. plantarum* L15 by using one-way analysis of variance, followed by Duncan's test.

Simulated gastrointestinal juices tolerance pattern of 15 strains was shown in Figure 1. All *Lactobacillus* strains are able to survive in the simulated gastrointestinal tract for 7 h and the survival rates ranged from 71.40 to 87.36%. It was also observed that *L. plantarum* L15 exhibited the highest survival rate with 87.36% (*P* < 0.05), following by *L. plantarum* L13, L17, L14, and L11.

**Figure 2:**
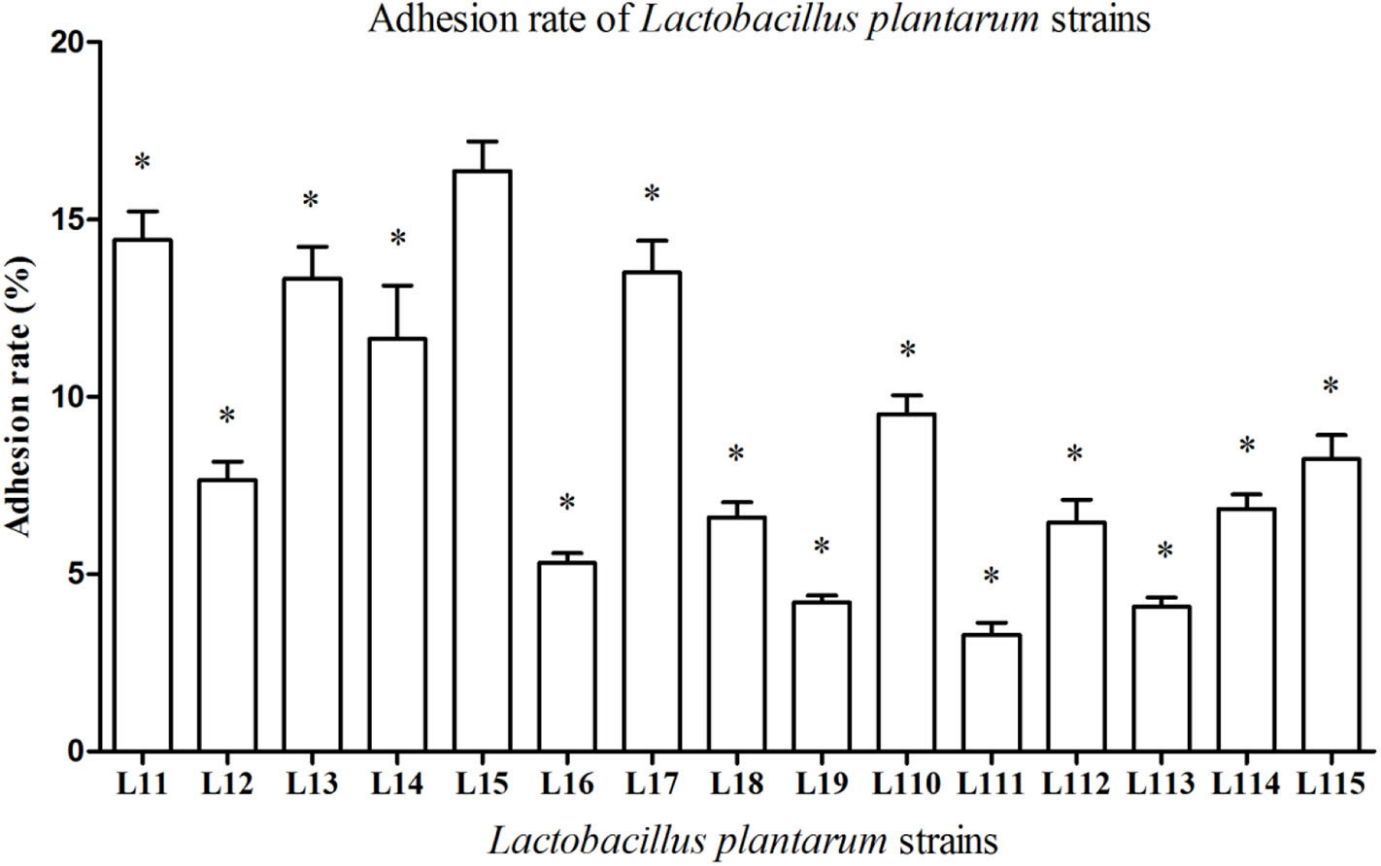
Adhesion rate of *Lactobacillus plantarum* strains to Caco-2 cells. Values are mean ± SD (*n* = 3 independent experiment). *P < 0.05: significantly different compared with *L. plantarum* L15 by using one-way analysis of variance, followed by Duncan's test.

All *Lactobacillus* strains showed adhesion to the Caco-2 cells, with adhesion rate ranging from 3.28 to 16.37% (Figure 2). The adhesion rate of *L. plantarum* L15 was markedly higher than other strains (*P* < 0.05), followed by *L. plantarum* L11, L17, L13, and L14. Therefore, *L. plantarum* L15, L11, L13, L14, and L17 strains had good *in vitro* probiotic properties, including high GIT tolerance and adhesion ability. They were selected to test the protective effect on the inflammatory activities induced by LPS in Caco-2 cells.

### Induction of Inflammatory Response in Caco-2 Cells

**Figure 3:**
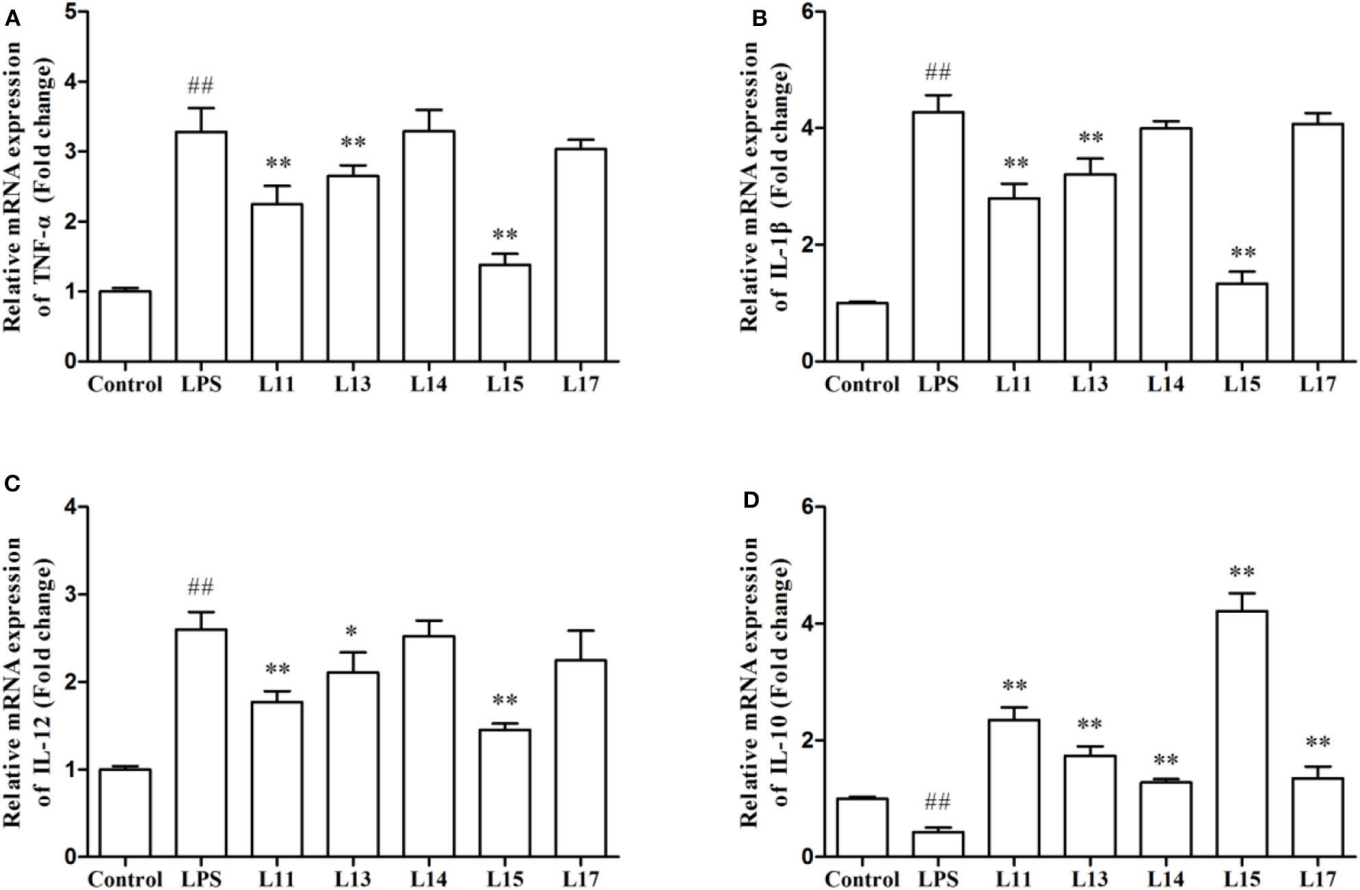
Effect of *Lactobacillus plantarum* strains on the expression levels of cytokine genes in Caco-2 cells. **(A)** TNF-α; **(B)** IL-1β; **(C)** IL-12; and **(D)** IL-10. Control, normal control group; LPS, LPS-induced inflammation group; L11, LPS plus *L. plantarum* L11; L13, LPS plus *L. plantarum* L13; L14, LPS plus *L. plantarum* L14; L15, LPS plus *L. plantarum* L15; L17, LPS plus *L. plantarum* L17. Values are mean ± SD (*n* = 6 independent experiment). ^##^*P* < 0.01: significantly different compared with the control group. *P < 0.05 and **P < 0.01: significantly different compared with the LPS group by using one-way analysis of variance, followed by Duncan's test.

The immune factor responses when Caco-2 cells were first induced with LPS solution and then treated with the selected *L. plantarum* strains, were determined in the present research (Figure 3). LPS remarkably stimulated the expression of TNF-α, IL-1β, and IL-12 genes, compared to the control (*P* < 0.01). After *L. plantarum* L15, L11, and L13 treatments, the expression levels of TNF-α, IL-1β, and IL-12 were significantly reduced (*P* < 0.05), whereas there were no significant changes observed in the *L. plantarum* L14 and L17 treatments (*P* > 0.05). Although all *L. plantarum* strains up-regulated the expression level of IL-10. However, treatment with *L. plantarum* L15 showed the highest IL-10 increase, suggesting its strong anti-inflammatory activity. It was found that *L. plantarum* L15 had strong reduction of pro-inflammatory effect by down-regulating the pro-inflammatory cytokine expression but up-regulating the anti-inflammatory cytokine expression in the Caco-2 cells. Based on these *in vitro* screening procedures, *L. plantarum* L15 was selected to investigate its *in vivo* anti-UC characteristics.

### Effect of *L. plantarum* L15 Supplementation on DSS-Induced Colitis in Mice

**Figure 4:**
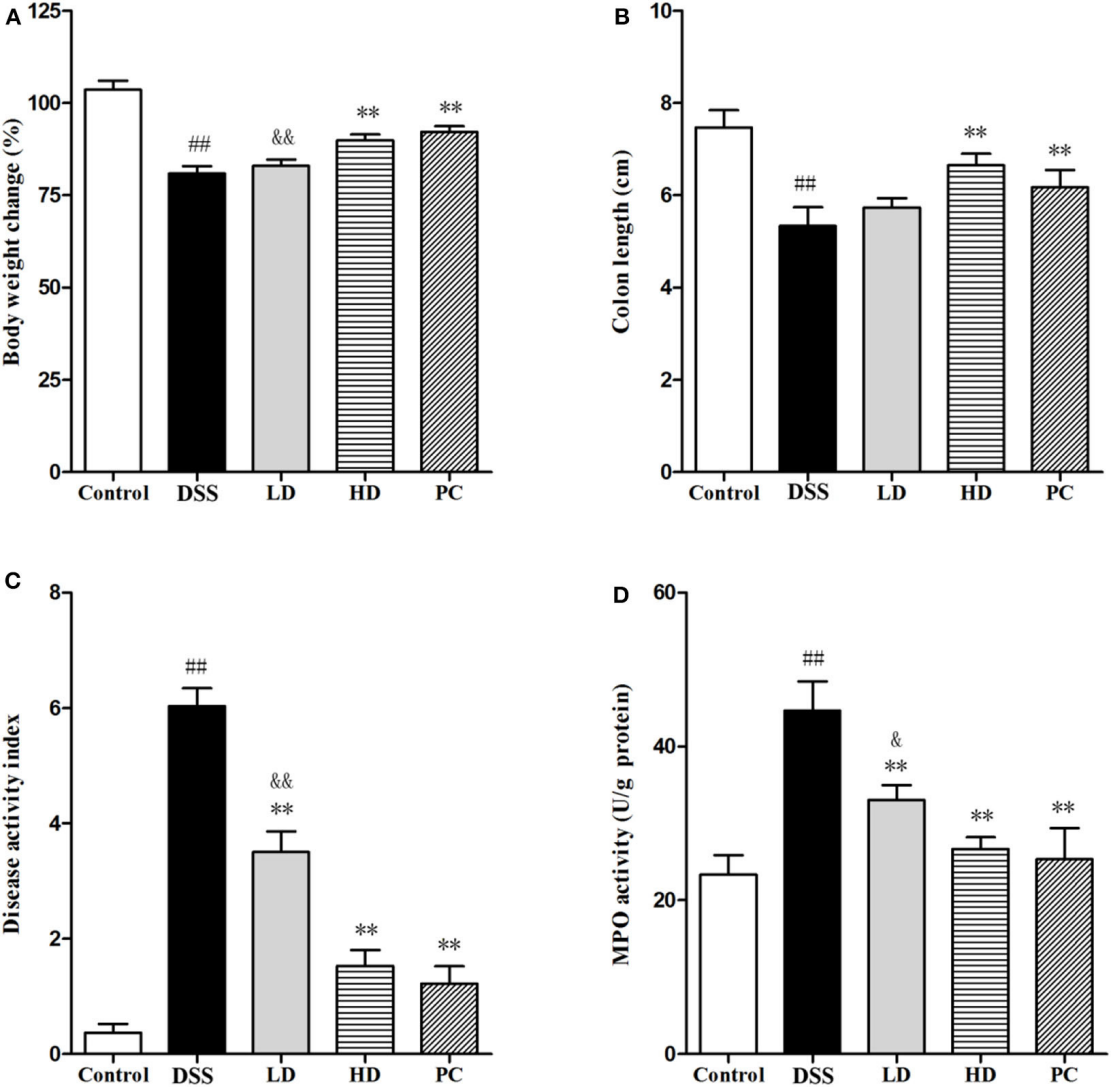
Effects of *L. plantarum* L15 on DSS-induced colitis in mice. **(A)** Body weight change (%); **(B)** Colon length; **(C)** DAI score; and **(D)** MPO activity. Control, normal control group; DSS, dextran sulfate sodium-induced colitis group; LD, DSS plus low dose of *L. plantarum* L15 (1 × 10^9^ CFU/mL, 1 mL/100 g body weight); HD, DSS plus high dose of *L. plantarum* L15 (1 × 10^10^ CFU/mL, 1 mL/100 g body weight). Values are mean ± SD (*n* = 6 independent experiment). ^##^*P* < 0.01: significantly different compared with the control group; **P < 0.01: significantly different compared with the DSS group by using one-way analysis of variance; ^&^*P* < 0.05 and ^&&^*P* < 0.01: significantly different compared with the PC group by using one-way analysis of variance, followed by Duncan's test.

As shown in Figure 4, DSS exposure significantly decreased the body weight and colon length (*P* < 0.01), but significantly increased the DAI and MPO when compared with the control group (*P* < 0.05). In the LD group, only the DAI and MPO significantly were reversed (*P* < 0.01), whereas, no significant changes of the body weight and colon length were observed (*P* > 0.05). All four indexes were significantly ameliorated in the HD group (*P* < 0.01), which were not significantly different with the PC group (*P* > 0.05). These results indicated that the high dose of *L. plantarum* L15 supplementation could effectively restore physiological changes in mice treated with DSS.

### Effect of *L. plantarum* L15 Supplementation on Cytokines Production

**Figure 5:**
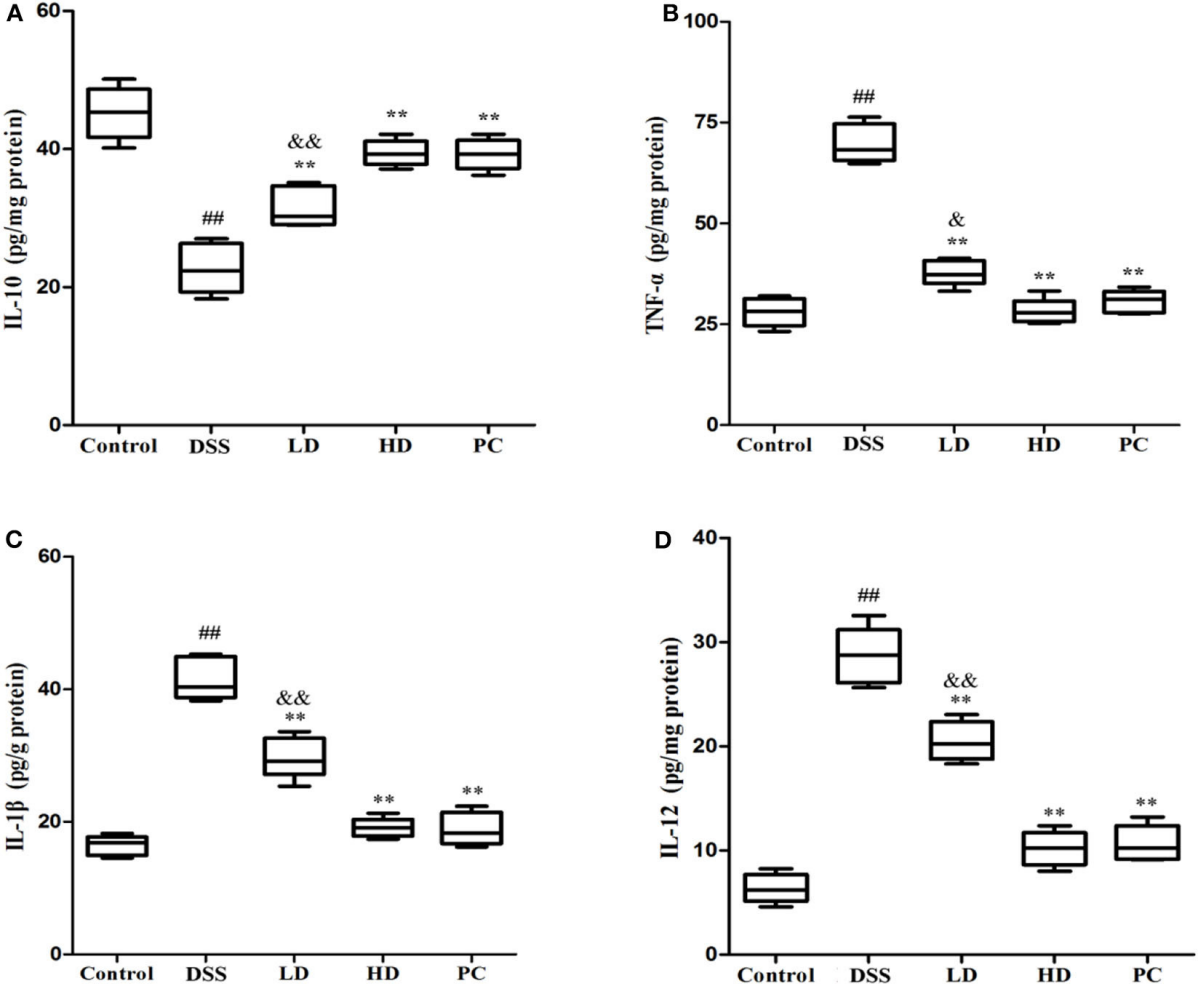
Effects of *L. plantarum* L15 on cytokines production (protein level) in colon tissues from DSS-induced colitis in mice determing by ELISAs. **(A)** IL-10; **(B)** TNF-α; **(C)** IL-1β; and **(D)** IL-12. Control, normal control group; DSS, dextran sulfate sodium-induced colitis group; LD, DSS plus low dose of *L. plantarum* L15 (1 × 10^9^ CFU/mL, 1 mL/100 g body weight); HD, DSS plus high dose of *L. plantarum* L15 (1 × 10^10^ CFU/mL, 1 mL/100 g body weight). Values are mean ± SD (*n* = 6 independent experiment). ^##^*P* < 0.01: significantly different compared with the control group; **P < 0.01: significantly different compared with the DSS group by using one-way analysis of variance; ^&^*P* < 0.05 and ^&&^*P* < 0.01: significantly different compared with the PC group by using one-way analysis of variance, followed by Duncan's test.

As shown in Figure 5, the IL-10 concentration in the DSS group was significantly lower than that in the control group, while the concentration of TNF-α, IL-1β, and IL-12 was significantly increased (*P* < 0.01), implying that DSS administration could increase the secretion of pro-inflammatory cytokine with a corresponding decrease in anti-inflammatory cytokine levels. The IL-10 concentration was conspicuously increased compared with the DSS group (*P* < 0.01) after low and high doses of *L. plantarum* L15 supplementation. It was also observed that TNF-α, IL-1β, and IL-12 were significantly decreased in these two supplementation groups compared to the DSS group (*P* < 0.01). More so, there were no significant differences of cytokine production between the HD and PC groups (*P* > 0.05). These results implied that high dose of *L. plantarum* L15 supplementation could more effectively alleviate UC by regulating Inflammatory cytokines.

### Effect of *L. plantarum* L15 Supplementation on Colon Injury Prevention

**Figure 6:**
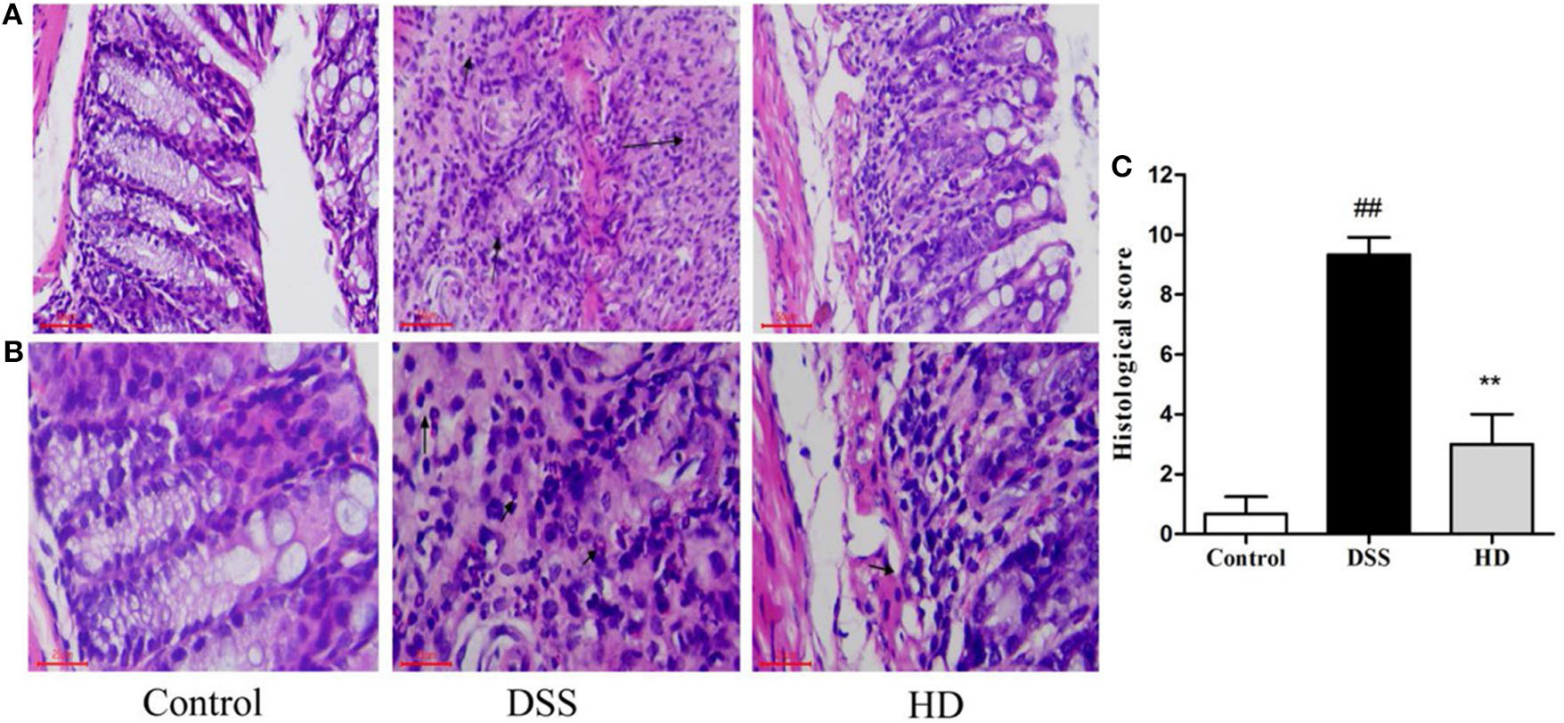
Effects of *L. plantarum* L15 on the colon histology. **(A)** Representative photomicrographs of colon histology (magnification 200×); **(B)** Representative photomicrographs of colon histology (magnification 400×) and **(C)** Associated histological scores. Control, normal control group; DSS, dextran sulfate sodium-induced colitis group; HD, DSS plus high dose of *L. plantarum* L15 (1 × 10^10^ CFU/mL, 1 mL/100 g body weight). Values are mean ± SD (*n* = 6 independent experiment). ^##^*P* < 0.01: significantly different compared with the control group; **P < 0.01: significantly different compared with the DSS group by using one-way analysis of variance, followed by Duncan's test.

To observe the effect of *L. plantarum* L15 supplementation on histopathological changes of UC mice, the HE staining of colonic tissue was performed. As presented in Figures 6A,B, the colon epithelial cells of mice in the control group were arranged in order, and there were abundant goblet cells without inflammatory cell infiltration. In the DSS group, colon edema was severe, epithelial structure was damaged, most of goblet cells were absent, and a noticeably high number of inflammatory cells infiltration. Compared to the DSS group, high dose of *L. plantarum* L15 supplementation attenuated the extent and severity of lesions, improved the epithelium architecture and reduced inflammatory cell infiltration. As shown in Figure 6C, the score value of the DSS group was significantly higher than that of the control group. In contrast, the score value of the HD group was lower than that of the DSS group. These results implied that high dose of *L. plantarum* L15 can alleviate and repair injuries caused in colon tissues by DSS induction.

### Effect of *L. plantarum* L15 Supplementation on Gut Microbiota Structure

Among the bacterial α-diversity, Chao 1, and Shannon indexes were used to estimate the richness and diversity of gut microbiota. As shown in Supplementary Figure 1A, the Chao 1 and Shannon indexes in the DSS group were significantly decreased when compared with the control group (*P* < 0.05). However, the two indexes in the HD group were significantly higher than that in the DSS group (*P* < 0.05). Among the bacterial β-diversity, the hierarchical clustering tree of weighted UniFrac distances showed that the HD group was grouped with the control group and then clustered with the DSS group (Supplementary Figure 1B). These findings indicated that high dose supplementation of *L. plantarum* L15 shifted the overall structure of the DSS mice gut microbiota to the control group.

**Figure 7:**
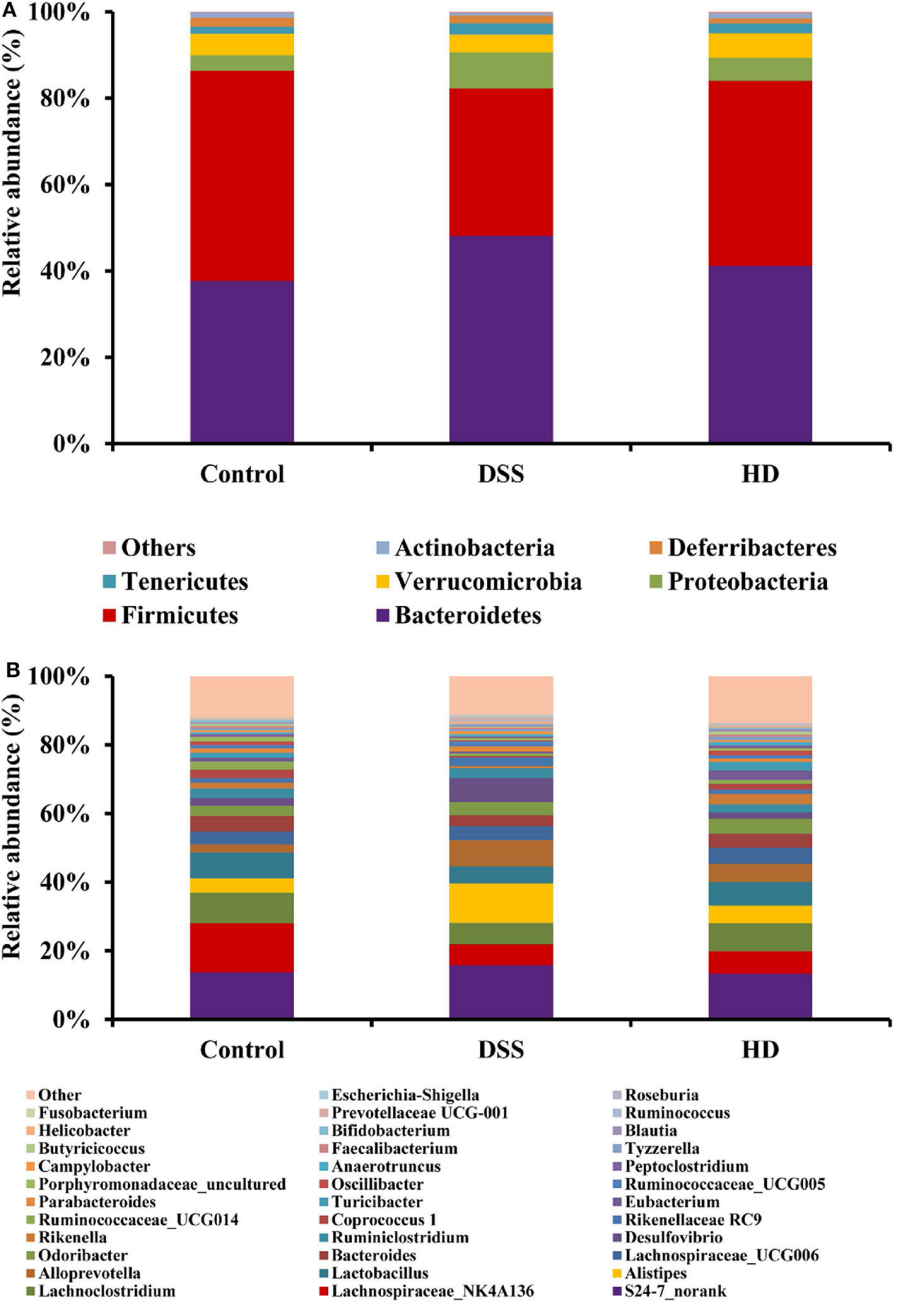
Relative abundance of gut microbiota at the phylum level **(A)** and genus **(B)** levels in the feces of mice. Control, normal control group; DSS, dextran sulfate sodium-induced colitis group; HD, DSS plus high dose of *L. plantarum* L15 (1 × 10^10^ CFU/mL, 1 mL/100 g body weight). Values are mean (*n* = 5 independent experiment).

The gut microbiota composition at the phyla and genera levels in three groups (control, DSS, and HD) shown in Figure 7. It was observed that the relative abundances of Bacteroidetes and Proteobacteria increased in the DSS group compared to the control, but the relative abundances of Actinobacteria and Firmicutes decreased (Figure 7A). After high dose supplementation with *L. plantarum* L15, the Firmicutes/Bacteroidetes ratio, and abundances of Proteobacteria and Actinobacteria were restored, thus suggesting its efficacy. At the genus level (Figure 7B), the abundances of *Lachnospiraceae_NK4A136* group, *Lactobacillus, Turicibacter, Bacteroides, Butyricicoccus, Bifidobacterium* and *Faecalibacterium* were reduced in the DSS group, confirming DSS induction. Interestingly, *L. plantarum* L15 supplementation normalized the disruptions in these genera. In addition, the relative abundances of other pathogenic genera such as *Alistipes, Alloprevotella, Desulfovibrio, Blautia, Odoribacter, Helicobacter, Parabacteroides, Campylobacter*, and *Escherichia-Shigella* increased in the DSS group, but inhibited by high doses of *L. plantarum* L15 supplementation.

**Figure 8:**
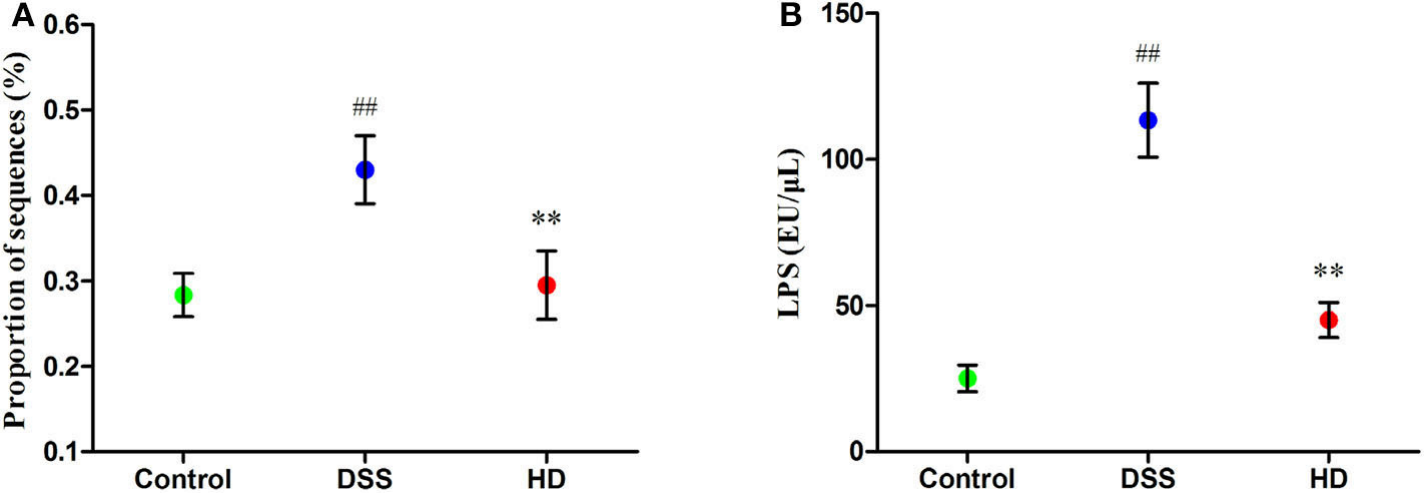
Function of LPS biosynthesis inferred from gut microbiota by PICRUSt annotation based on 16S rRNA gene sequences **(A)** and the LPS level in the cecal contents **(B)**. Control, normal control group; DSS, dextran sulfate sodium-induced colitis group; HD, DSS plus high dose of *L. plantarum* L15 (1 × 10^10^ CFU/mL, 1 mL/100 g body weight). Values are mean (*n* = 5 independent experiment). ^##^*P* < 0.01: significantly different compared with the control group; **P < 0.01: significantly different compared with the DSS group by using one-way analysis of variance, followed by Duncan's test.

The abundances of functional category of LPS biosynthesis in the KEGG pathway was shown in Figure 8A. Compared to the control group, the microbial gene associated with LPS biosynthesis significantly increased in the DSS group (*P* < 0.01), but this pattern was reversed by *L. plantarum* L15 supplementation. As shown in Figure 8B, the concentration of LPS in the cecal contents in the HD group were significantly lower than that in the DSS group (*P* < 0.01).

### Effect of *L. plantarum* L15 Supplementation on the Expression of NF-κB

**Figure 9:**
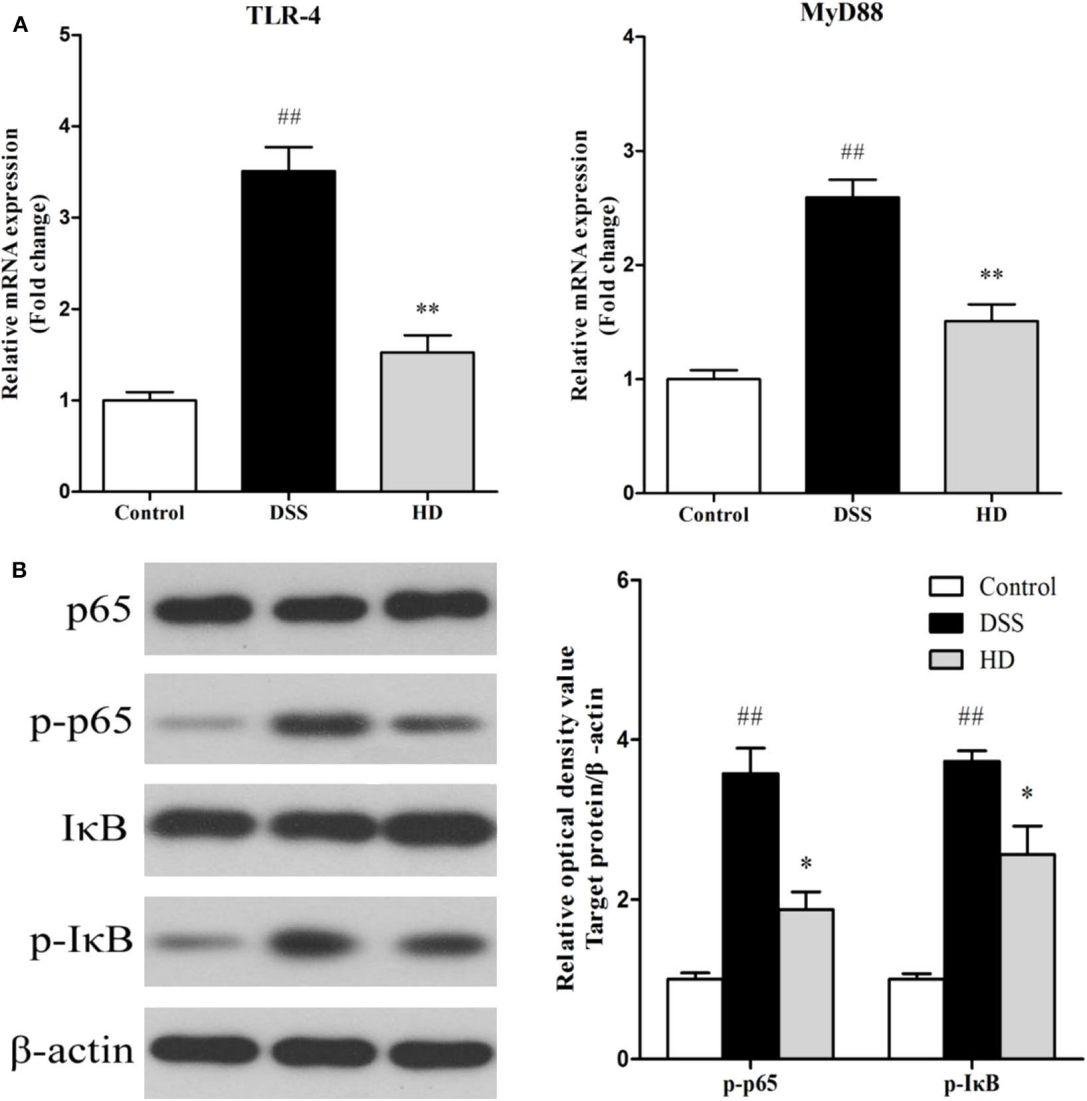
The mRNA levels of TLR4 and MyD88 **(A)** and the protein levels of NF-κB signaling pathway in colon tissue **(B)**. Control, normal control group; DSS, dextran sulfate sodium-induced colitis group; HD, DSS plus high dose of *L. plantarum* L15 (1 × 10^10^ CFU/mL, 1 mL/100 g body weight). Values are mean (*n* = 6 independent experiment). ^##^*P* < 0.01: significantly different compared with the control group. *P < 0.05 and **P < 0.01: significantly different compared with the DSS group by using one-way analysis of variance, followed by Duncan's test.

The effects of *L. plantarum* L15 supplementation on the colonic TLR4 and MyD88 expression genes at mRNA level are shown in Figure 9A. Compared with the control group, TLR4, and MyD88 genes in the DSS group were significantly (*P* < 0.01) up-regulated, while *L. plantarum* L15 administration significantly down-regulated their expression levels (*P* < 0.01), when compared with the DSS group. Expression of genes involved in NF-κB signaling pathway in the colonic tissue at protein level determined by western-blot are shown in Figure 9B. DSS induction resulted in a significant increase in the expression of p-p65 and p-IκB factors as compared to the control group (*P* < 0.01). Interestingly, *L. plantarum* L15 supplementation significantly reduced the expression of p-p65 and p-IκB (*P* < 0.05). These results indicated that *L. plantarum* L15 supplementation could attenuate chemical-induced colitis by DSS through inhibiting the TLR4-MyD88-NF-κB signaling pathways.

## Discussion

Inflammatory bowel disease (IBD), including ulcerative colitis (UC) and Crohn's Disease (CD), is a global health challenge that is currently on the rise (2, 40). According to several epidemiology and micro-ecology studies, findings suggest that its pathogenic mechanism is related to deregulation of the host immune response and intestinal micro-ecosystem (6). It is thus proposed by many researchers that improving the gut microbiota and boosting the immune response, can be safe and promising strategies for the patients suffering from UC (2).

Previous studies have indicated the possible link between activities of *Lactobacillus* strains and enhancement of host immunity. Generally, different *Lactobacillus* strains may exert positive or negative effects on the UC severity *in vivo*. This diversity is strain-specific and closely related to several key factors. GIT tolerance and Caco-2 cell adhesion characteristics are widely used *in vitro* screening procedure to ascertain potential probiotic strains with possible beneficial intestinal functions as they are associated with the survival of a strain and its colonization in the intestine (41). Furthermore, while good tolerance levels are indicative of the ecological resilience of a given strain (42), its adhesion ability can be a good predictor of immune modulation enhancement and maintenance gut barrier integrity (43). The present study selected potential probiotic strains from 15 *L. plantarum* strains *in vitro* based on these two procedures. Based on the *in vitro* results, *L. plantarum* L15 was selected for further investigations *in vivo* using a DSS-induced UC model.

DAI and MPO are widely used to evaluate UC condition (44, 45). DAI is composed of body weight change, diarrhea, and hematochezia, which are measured every day. It plays an important role in evaluating the clinical progression of UC (46). It has been reported that MPO is a simple, non-invasive, and relevant marker of UC activity (47). In present study, the DAI and MPO significantly increased in the DSS group, indicating that the UC model was successfully constructed. The two indexes were reversed by the high dose of *L. plantarum* L15 supplementation. These results indicated that *L. plantarum* L15 supplementation could effectively restore physiological changes in mice treated with DSS.

Cytokines are protein substances that mediate immunity and inflammation activities. One type of cytokine, the TNF-α has been linked with pro-inflammatory responses. Similarly, another category of cytokines secreted from macrophages, the IL-1β, is involved in pro-inflammatory regulations that facilitates UC progression (48). In addition, T and natural killer cells are mediated by the IL-12 cytokine, which in turn fuels TNF-α secretion (49). As reported previously, pro-inflammatory cytokine levels (TNF-α, IL-12, and IL-1β) in colonic tissues were elevated significantly by DSS exposure (50, 51). These cytokines have been inhibited by *Lactobacillus* species, endowing the latter with reduction of pro-inflammatory properties (52–54). Results from the current study showed that pro-inflammatory cytokine levels (TNF-α, IL-1β, and IL-12) in the DSS group were significantly higher compared to the control group but these anomalies were also significantly reversed by high-dose administration of *L. plantarum* L15. Furthermore, the amounts of an anti-inflammatory cytokine, IL-10, was markedly increased by this probiotic supplementation. These observations posited that a high-dose administration of *L. plantarum* L15 had UC-alleviating effects and thus could be a potential candidate in UC therapy.

The gut ecosystem is a diverse collection of bacteria genera that play specific and inter-related roles in host immunity, disease prevention, and beneficial intestinal functions. This makes the gut microbiota a key research area in many intervention studies. It is no surprise therefore that an unstable gut ecology was previously linked with the onset of many diseases, including UC and CD (55, 56). Observations from the present study showed that the relative abundances of the Bacteriodetes and Proteobaceria phyla increased in the DSS group compared with the control group, agrees with previous reports (20).

At the genus level, the distribution varied in all studied groups. The relative abundances of *Lachnospiraceae._NK4A136* group, *Lactobacillus, Turicibacter, Bacteroides, Butyricicoccus, Bifidobacterium*, and *Faecalibacterium* were reduced in DSS-induced mice but increased after high dose of *L. plantarum* L15 administration. The *Lactobacillus* genera is a well-known probiotic group with colitis-alleviating effects in *in vivo* mouse models (45, 57). Samples from IBD patients indicate that the relative abundance of the *Butyricicoccus* genera was reduced (58). This genera is known to improve colitis status by inhibiting the NF-κB transcription factor in lamina propria macrophages of UC patients (59, 60). Another genera, *Bifidobacterium*, is an extensively-used probiotic group and it was previously reported that its consumption could alleviate inflammations in mice intestine, thus inferring that it could play beneficial roles in UC management (61, 62). In addition to these, activities of the *Faecalibacterium* genera have been linked in intestinal health integrity and that the relative abundance of *F. prausnitzii* was significantly reduced according to an earlier study involving UC patients (10).

In contrast, up-regulation of some pathogenic genera like *Alistipes, Alloprevotella, Desulfovibrio, Blautia, Odoribacter, Helicobacter, Parabacteroides, Campylobacter*, and *Escherichia-Shigella* was noticed in the DSS group, which was suppressed by high-dose *L. plantarum* L15 administration. The *Bacteroides, Alistipes, Parabacteroides* and *Alloprevotella* genera belong to the Bacteroidetes phylum, and are known for LPS secretion (63). In addition, a previous research reported that two gastrointestinal pathogens, *Helicobacter. pylori* and *Campylobacter jejuni* not only belong to the epsilon-proteobacteria class, but also possess specific lipid A structures which implicates them in LPS-related infections. These researchers also reported that the proteobacterium pathogen, *Desulfovubrio desulfuricans* could secrete LPS, which could exacerbate UC and CD conditions (64). Furthermore, elevated *E. coli* levels have been reported in UC sufferers and *in vivo* mouse models (65). Cani et al. confirmed previous postulations by demonstrating that a significant portion of these gram-negative pathogenic bacteria membranes are composed of LPS, and this explains why they are endowed with the propensity for pro-inflammatory activities in the gastrointestinal tract and promote gut dysbiosis (66).

Previously, Ning et al. postulated that the TLR4 receptor is triggered by LPS activities, and that the former has been linked with low-grade chronic inflammatory diseases (67). Inflammatory cytokines secretion are activated by the TLR4/NF-κB complex, formed upon successful binding of the TLR4 receptor and LPS (68). The NF-κB transcription factor has been shown to activate key genes involved in pro-inflammatory cytokine production, which lower the body's defense system over time. These pathways and processes eventually contribute to UC onset (69, 70). Thus, we further assessed the TLR4 -NF-κB signaling pathway. In the present study, the expression of TLR4, p-p65, and p-IκB was higher in the DSS group than that in the control group, but this trend was reversed by high dose of *L. plantarum* L15 administration. In addition, TLR4 up-regulation has been detected during intestinal examination of IBD patients, its activation by DSS administration has been reported (71, 72).

## Conclusion

In summary, *in vitro* and *in vivo* results from our study demonstrated that *L. plantarum* L15 possessed good gastrointestinal transit tolerance, adhesion, and reduction of pro-inflammatory abilities. In addition, the gut microbiota of UC mice were positively regulated by high dose of *L. plantarum* L15 supplementation, as evidenced in down-regulating LPS levels, which in turn suppressed the activation of the TLR4-NF-κB signaling pathway.

## Data Availability Statement

The datasets presented in this study can be found in online repositories. The names of the repository/repositories and accession number(s) can be found at: https://www.ncbi.nlm.nih.gov/, PRJNA4501.

## Ethics Statement

The animal study was reviewed and approved by the Institutional Animal Care and Use Committee of the Northeast Agricultural University.

## Author Contributions

BL and XZ conceived the study and designed the project. PY and CK performed the experiment, analyzed the data, and drafted the manuscript. JG helped to revise the manuscript. All authors read and approved the final manuscript.

## Conflict of Interest

The authors declare that the research was conducted in the absence of any commercial or financial relationships that could be construed as a potential conflict of interest.
