# Supplementary material for: Lactobacillus plantarum L15 Alleviates Colitis by Inhibiting LPS-Mediated NF-κB Activation and Ameliorates DSS-Induced Gut Microbiota Dysbiosis
Source: Front Immunol. 2020 Oct 2;11:575173. doi: 10.3389/fimmu.2020.575173 (PMC7566170; doi:10.3389/fimmu.2020.575173)
Supplement: Supplementary file 1 [file Data_Sheet_1.docx]

**Table S1** Primer sequences of mRNA for the expression of cytokine genes through RT-PCR.

| Genes | Primer sequence (5'-3') |
| --- | --- |
| GAPDH | F: AACGGATTTGGTCGTATTG |
|  | R: GCTCCTGGAAGATGGTGAT |
| TNF-α | F: CCCAGGGACCTCTCTCTAATC |
|  | R: ATGGGCTACAGGCTTGTCACT |
| IL-1β | F: GTGGCAATGAGGATGACTTGTTC |
|  | R: TTGCTGTAGTGGTCGGAG |
| IL-12 | F: GAGGCCTGTTTACCATTGGA |
|  | R: TACTAAGGCACAGGGCCATC |
| IL-10 | F: TCAGGGTGGCGACTCTAT |
|  | R: TGGGCTTCTTCTAAATCGTTC |

**Table S2** Scoring criteria for DAI.

| Score | Body weight loss | Stool consistency |
| --- | --- | --- |
| 0 | None | Normal |
| 1 | 1-5% | - |
| 2 | 5-10% | Loose stools, hemoccult positive |
| 3 | 10-20% | - |
| 4 | ＞20% | Diarrhea, gross bleeding |

**Table S3** Raw reads data of 16S rDNA sequencing.

| Group | Raw reads |
| --- | --- |
| Control1 | 88020 |
| Control2 | 89716 |
| Control3 | 140342 |
| Control4 | 89572 |
| Control5 | 112338 |
| DSS1 | 140086 |
| DSS2 | 90414 |
| DSS3 | 143444 |
| DSS4 | 106000 |
| DSS5 | 116202 |
| HD1 | 107926 |
| HD2 | 90564 |
| HD3 | 116812 |
| HD4 | 92568 |
| HD5 | 89852 |

**Table S4** Primer sequences of mRNA for the expression of TLR4 and MyD88 genes through RT-PCR.

| Genes | Primer sequence (5'-3') |
| --- | --- |
| β-actin | F: TGCTGTCCCTGTATGCCTCT |
|  | R: AGGTCTTTACGGATGTCAACG |
| TLR4 | F: GCTCTCAGCCATCCACAAAG |
|  | R: GAGTCGGGAAGAGGAAGAGG |
| MyD88 | F: CTAAGAAGGACCAGCAGAG |
|  | R: GAAGCATCAGTAGGCATCA |


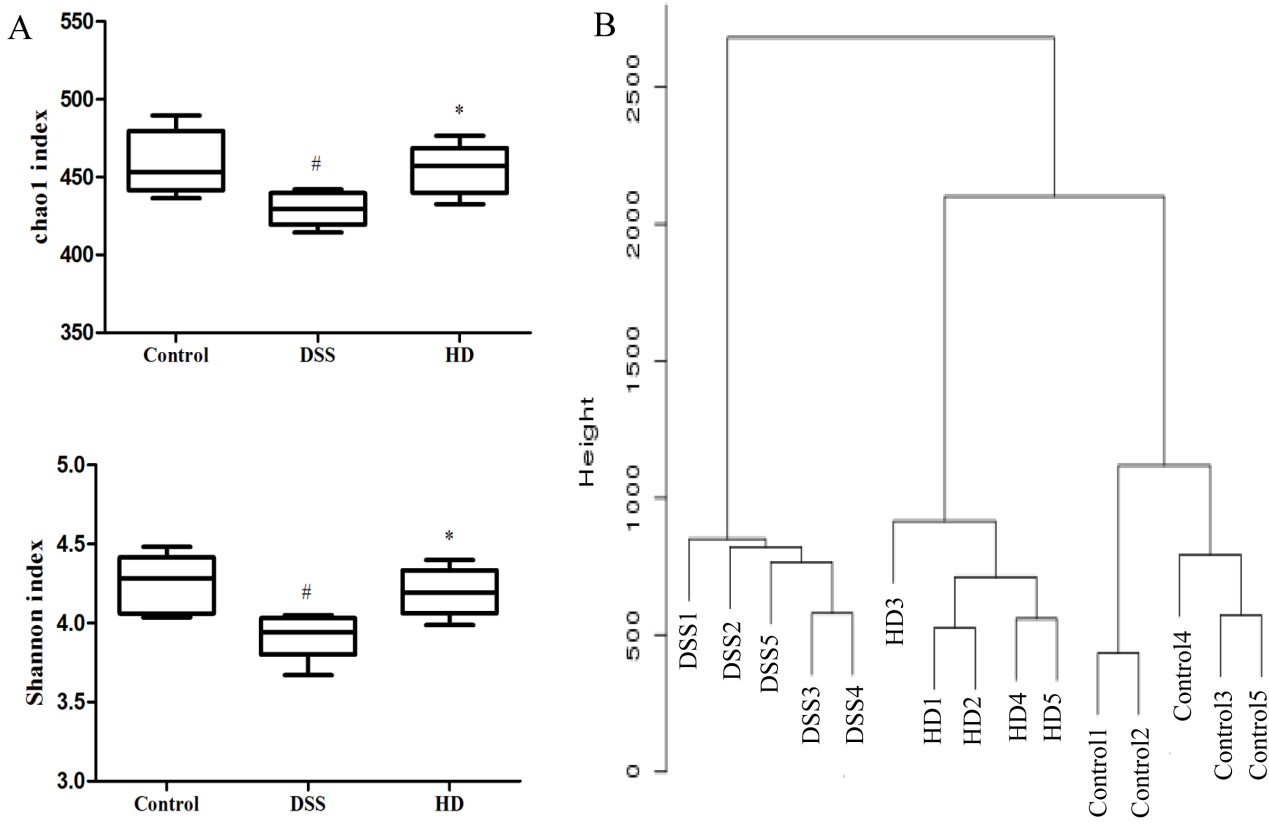


**Figure S1** The effect of *L. plantarum* L15 on gut microbiota α-diversity and β-diversity. (**A**) Chao 1 and Shannon index; (**B)** Hierarchical clustering tree of weighted UniFrac distances. Control, normal control group; DSS, dextran sulfate sodium-induced colitis group; HD, DSS plus high dose of *L. plantarum* L15 (1 × 10^10^ CFU/mL,1 mL/100 g body weight). Values are mean ± SD (n=5 independent experiment). ^#^ *P* < 0.05 and ^##^ *P* < 0.01: significantly different compared with the control group; * *P* < 0.05 and ** *P* < 0.01: significantly different compared with the DSS group by using one-way analysis of variance, followed by Duncan’s test.
